# Alcohol at Certain General and Special Metropolitan Hospitals, 1875-6 to 1885

**Published:** 1886-10-30

**Authors:** 


### Hospital Administration.

[Continued from page 41.—Communications for this column should be addressed, "Administrator," care of the Editor of The Hospital.]

### ALCOHOL AT CERTAIN GENERAL AND SPECIAL METROPOLITAN HOSPITALS, 1875-6 to 1885.

**Explanatory.** In further elucidation of the facts in relation to the consumption of alcohol at the metropolitan hospitals, it has been found necessary to divide the general and special into two classes: (*a*) Those general and special hospitals whose books give detailed returns, showing the number of the staff and of the in-patients each year, and the amount of spirits of wine consumed, in addition to malt liquors, wines, and spirits; and (*b*) those institutions where figures were not attainable in such detail.

To-day we deal with the second group, which includes the five general and fourteen special hospitals named in the following Table. Space does not permit us to give the number of patients under treatment in the two years referred to, and it will be seen that very few of these institutions make any return as to the cost of the spirits of wine consumed. There were, however, approximately, 11,295 in-patients treated at the under-mentioned hospitals in 1875-6, and 13,555 in-patients in 1885.

**Differences in Systems of Diet.** The effect of the removal of malt liquors and all stimulants from the diet-table during recent years is clearly shown in the figures given below. Thus, at the Charing Cross Hospital the reduction in the consumption of malt liquors in the year 1885, as compared with 1875, although the patients under treatment increased nearly 100 per cent., is not a little remarkable, and the same may be said of the special hospitals, where details are given. This alteration of system has not materially reduced the expenditure in the case of these hospitals any more than it has done at the London Hospital, as we proved a fortnight ago, and for the same reasons. Thus, although the total amount spent on alcohol fell from £4,781 in 1875 to £3,310 in 1885, the cost of milk increased from £3,888 in 1875 to £5,269 in 1885. The reduction in the expenditure upon alcohol means, therefore, an equal, and in some cases a greater, expenditure upon milk.

**The Actual Reduction in Alcohol.** At the five general hospitals, the expenditure on alcohol was £3,033 in 1875, when the number of in-patients was 7,933, against £2,233 in 1885, when the in-patients had increased to 8,996. This reduction is the more remarkable because the alcohol expenditure for the year 1885 includes £280, the cost of spirits of wine consumed at St. Mary's and the Middlesex Hospitals, an item which is not included in the total for 1875. At the fourteen special hospitals, £1,757 was spent upon alcohol in 1875, against £1,076 spent in 1885, although 1,200 more in-patients were treated in the latter as compared with the former year. It appears, therefore, that the expenditure on alcohol at the nineteen special and general hospitals given in the Table amount to 8*s*. 4½*d*. per in-patient in 1875, and to only 4*s*. 10½*d*. in 1885. These figures are not precisely accurate, as they include cost of alcohol consumed by the staff, it being impossible to separate this item from the general total.

*Value of the Consumption of Milk and of Alcohol (so far as ascertainable) at five General and fourteen Special Hospitals during the years 1875 and 1885 respectively.*

| | 1875. | | | | | 1885. | | | | |
|---|---|---|---|---|---|---|---|---|---|---|
| | Milk. | Wine and Spirits. | Malt Liquors for Staff. | Malt Liquors for Patients. | Total of Alcohol. | Milk. | Wine and Spirits. | Malt Liquors for Staff. | Malt Liquors for Patients. | Total of Alcohol. |
| | £ s. d. | £ s. d. | £ s. d. | £ s. d. | £ s. d. | £ s. d. | £ s. d. | £ s. d. | £ s. d. | £ s. d. |
| Charing Cross Hospital .. | 412 15 2 | 590 8 2 | 72 15 6 | 120 5 1 | 783 8 9 | 547 8 6 | 302 4 11 | 34 14 8 | 50 5 0 | 387 4 7 |
| Poplar Hospital .. .. | 142 0 0 | 27 19 0 | .... | 83 3 0 | 111 2 0 | 94 16 5 | 48 19 0 | 57 6 10 | .... | 106 5 10 |
| St. Mary's Hospital .. | 312 0 10 | 249 15 9 | .... | 185 11 5 | 435 7 2 | 931 8 11 | 328 0 10 | .... | 157 9 9 | *649 3 8 |
| Seamen's Hospital Society .. | 578 0 0 | 449 0 0 | .... | 195 0 0 | 644 0 0 | 491 0 0 | 248 0 0 | .... | 166 0 0 | 414 0 0 |
| Middlesex Hospital .. | 924 19 0 | 516 7 6 | .... | 532 15 8 | 1049 3 2 | 915 2 0 | 242 11 0 | 222 17 7 | 95 1 5 | †676 7 6 |
| 5 General Hospitals .. | 2369 15 0 | 1833 10 5 | 72 15 6 | 1116 15 2 | 3023 1 1 | 2979 15 10 | 1169 15 9 | 314 19 1 | 468 16 2 | 2233‡ 1 7 |
| North London Hospital for Consumption | 106 4 11 | 35 12 10 | 52 6 3 | .... | 87 19 1 | 182 0 10 | 35 16 5 | .... | 12 12 0 | 48 8 5 |
| Alexandra Hospital .. | 120 17 8 | 25 2 8 | 31 3 3 | .... | 56 5 11 | 140 18 1 | 3 5 9 | 2 8 2 | .... | 5 13 11 |
| Belgrave Hospital for Children | 48 12 7 | 10 19 0 | 20 14 10 | .... | 31 13 10 | 50 17 4 | 8 13 8 | 18 7 10 | .... | 27 1 6 |
| Evelina Hospital.. .. | 170 0 0 | 60 15 3 | 6 0 0 | .... | 66 15 3 | 225 5 0 | 23 2 0 | 46 15 0 | .... | 69 17 0 |
| Victoria Hospital for Children | 54 2 0 | 6 16 6 | 14 17 0 | .... | 21 13 6 | 162 1 6 | 17 17 5 | 8 11 0 | .... | 26 8 5 |
| British Lying-In Hospital .. | 32 0 0 | 16 0 0 | 14 0 0 | 9 0 0 | 39 0 0 | 37 0 0 | 14 0 0 | 16 0 0 | 11 0 0 | 41 0 0 |
| City of London Lying-In Hospital | 29 7 5 | 36 6 5 | 51 3 6 | 72 2 0 | 159 11 11 | 53 6 11 | 32 16 8 | 12 14 2 | 12 19 0 | 58 9 10 |
| Chelsea Hospital for Women.. | 41 15 10 | 4 15 0 | .... | .... | 4 15 0 | 173 10 1 | 23 8 0 | 17 2 0 | .... | 40 10 0 |
| Hospital for Women .. | 164 11 6 | 45 19 7 | .... | 86 9 0 | 132 8 7 | 276 7 3 | 111 19 5 | 32 2 4 | .... | 144 1 9 |
| Cancer Hospital .. .. | 90 0 0 | 359 0 0 | .... | 186 0 0 | 545 0 0 | 272 0 0 | 155 0 0 | .... | 168 0 0 | 323 0 0 |
| London Fever Hospital .. | 277 12 9 | 201 18 0 | .... | 112 4 10 | 314 2 10 | 290 15 11 | 72 15 6 | .... | 85 18 2 | 158 13 8 |
| St. Mark's Hospital for Fistula | 78 2 1 | 38 12 0 | .... | 35 9 10 | 74 1 10 | 103 19 3 | 16 1 9 | .... | 5 10 0 | 21 11 9 |
| Bournemouth Sanatorium .. | 266 15 5 | 54 10 0 | .... | 108 7 9 | 162 17 9 | 239 0 0 | 21 19 3 | 69 10 0 | .... | 91 9 3 |
| Invalid Asylum .. .. | 38 2 9 | 40 2 0 | .... | 21 1 6 | 61 3 6 | 82 8 10 | 9 2 6 | .... | 11 1 3 | 20 3 9 |
| 14 Special Hospitals .. | 1518 4 11 | 936 9 3 | 190 4 10 | 630 14 11 | 1757 9 0 | 2289 11 0 | 545 18 4 | 223 10 6 | 307 0 5 | 1076 9 3 |
| Total of 5 General and 14 Special Hospitals | 3887 19 11 | 2769 19 8 | 263 0 4 | 1747 10 1 | 4780 10 1 | 5269 6 10 | 1715 14 1 | 538 9 7 | 775 16 7 | 3309‡10 10 |

* Including £163 13*s*. 1*d*. cost of Spirits of Wine. † Including £115 17*s*. 6*d*. cost of Spirits of Wine.

‡ Including £279 10*s*. 7*d*. cost of Spirits of Wine.

